# Bioinformatics Analyses of Potential miRNA-mRNA Regulatory Axis in HBV-related Hepatocellular Carcinoma

**DOI:** 10.7150/ijms.50126

**Published:** 2021-01-01

**Authors:** Dan-Ping Huang, Yi-Hao Zeng, Wei-Qu Yuan, Xiu-Fang Huang, Sheng-Qian Chen, Mu-Yao Wang, Yi-Jun Qiu, Guang-Dong Tong

**Affiliations:** 1 Department of Hepatology, Shenzhen Traditional Chinese Medicine Hospital, The Fourth Clinical Medical College of Guangzhou University of Chinese Medicine, Shenzhen 518033, Guangdong Province, China.; 2 College of Basic Medicine, Guangzhou University of Chinese Medicine, Guangzhou, Guangdong, China.; 3 Department of Acupuncture, Shenzhen Traditional Chinese Medicine Hospital, The Fourth Clinical Medical College of Guangzhou University of Chinese Medicine, Shenzhen 518033, Guangdong Province, China.; 4 The First Affiliated Hospital of Guangzhou University of Chinese Medicine, Guangzhou 510403, Guangdong Province, China.; 5 Traditional Chinese Medicine Hospital of Haifeng County, Shanwei 516400, Guangdong Province, China.

**Keywords:** Hepatocellular carcinoma, microRNAs (miRNAs), Hepatitis B virus (HBV), bioinformatic analysis

## Abstract

**Aims:** We aimed to explore the crucial miRNA-mRNA axis through bioinformatics analysis and provide evidences for the development of pathophysiological mechanisms and new therapies for HBV-related HCC.

**Methods:** MiRNA (GSE76903) and mRNA (GSE77509) dataset were used to screen differentially expressed miRNAs (DE-miRNAs) and differentially expressed mRNAs (DE-mRNAs) using R software. Overlapping genes between DE-mRNAs and target genes of DE-miRNAs were identified as candidate genes. Hub genes were obtained via cytohubba analysis. The expression at protein and mRNA levels and prognostic value of hub genes were evaluated based on The Cancer Genome Atlas (TCGA) data. Key miRNA-mRNA axes were constructed according to predicted miRNA-mRNA pairs. MiRNA expression and prognostic role were respectively identified using starBase v3.0 and Kaplan-Meier plotter database. Real-time PCR was performed to verify the expression of crucial miRNAs and mRNAs. Coexpression of crucial miRNA and mRNA were analyzed using starBase v3.0.

**Results:** *CDK1, CCNB1, CKS2* and *CCNE1* were screened as hub genes, which were significantly upregulated at protein and mRNA levels. These up-regulated hub genes were also significantly associated with poor prognosis. Hsa-mir-195-5p/*CDK1*, hsa-mir-5589-3p/*CCNB1* and hsa-let-7c-3p/*CKS2* were screened as critical miRNA-mRNA axes. Critical miRNAs were decreased in HCC, which indicates unfavourable prognosis. QPCR results showed that crucial miRNAs were decreased, whereas critical mRNAs were increased in HBV-related HCC. A reverse relationship between miRNA and mRNA in crucial axis was further verified.

**Conclusion:** This study identified several miRNA-mRNA axes in HBV-related HCC. Hsa-mir-195-5p/*CDK1*, hsa-mir-5589-3p/*CCNB1* and hsa-let-7c-3p/*CKS2* might serve as potential prognostic biomarkers and therapeutic targets for HBV-related HCC.

## 1. Introduction

Hepatocellular carcinoma (HCC) is one of the most prevalent malignancies and the second most common cause of cancer-related death 1. Studies show that risk factors for HCC include hepatitis B virus (HBV) infection, hepatitis C virus (HCV) infection, parasite, alcohol abuse, excess cigarette smoking, autoimmune hepatitis, and so on 2. It is worth noting that 70% of HCC is caused by chronic HBV infection 3. Treatment strategies such as surgical resection, chemotherapy, radiotherapy, and liver transplantation have been applied on patients with HCC nowadays. However, HCC is highly resistant to existing treatments and the overall survival chance for patients with HCC is as low as other solid tumors (5-year survival rate <20%) due to genetic and epigenetic heterogeneity 4. Therefore, the molecular mechanism of the occurrence, progression and metastasis of HBV-related HCC need to be studied in depth so as to develop diagnosis of better accuracy, discover prognostic biomarkers of novelty and explore treatment strategies of effectiveness for HBV-related HCC.

MicroRNAs (miRNAs) are categorized as a class of small endogenous noncoding RNAs with 21-25 nucleotides 5. MiRNAs act as RNA silencing and post-transcriptional regulators and play pivotal roles in messenger RNAs (mRNAs)' degradation and translation suppression through base-paired binding at complementary sequences of mRNAs 6. Recent evidences demonstrate that dyregulation of miRNA is involved in the susceptibility, development and outcome of HCC. For example, Hung et al. extract the RNA from the serum of 30 chronic HBV-infected patients with dysplastic nodule and 120 patients with early HCC. They find that sensitivity of circulating miR-122 and let-7 is comparable to AFP testing, which can be reasonably hypothesized that they may be the useful markers for diagnosis of early HCC and HBV infection with dysplastic nodule 7. Let-7f is also significantly overexpressed in patients with a tumor size above 5 cm in diameter and early recurrence 8. MiR-148a, which is downregulated by the HBx protein, fails to suppress oncogenic expression involved in the PI3/MAPK pathway 9, and miR-125b-5p and miR223-3p are recognized as potential non-invasive biomarkers in HBV-related HCC through plasma analysis 10. Although researches on miRNAs of HBV-related HCC have made considerable progress, potential miRNA/mRNA pairs are still needed to be found to reveal the molecular mechanism of carcinogenesis and development for HBV-related HCC.

Recently, bioinformatics analysis has been widely used to study potential molecular mechanisms and therapeutic targets of a variety of diseases. An increasing number of miRNA/mRNA pairs are found to play crucial roles in various cancer processes through high-throughput microarray and bioinformatics analysis. Hou et al. carry out an in-depth analysis of miRNomes in human HCC, finding that miR-199a/b-3p might supress HCC growth through targeting *PAK4* 11. Lou et al. conduct an integrated analysis of miRNA-mRNA regulatory pathways for the pathogenesis of HBV-related HCC and screen six candidate target genes via qRT-PCR and expression correlation analysis 12. However, the miRNA and mRNA data of HBV-related HCC in previous studies were usually selected from different clinical samples, which might lack rigor in data screening, let alone limited supporting evidence of identified genes in available research. Therefore, it is still of great necessary to find more novel miRNA/mRNA pairs from the same clinical samples in order to provide more potential approaches for study on HBV-related HCC.

In this study, miRNA and mRNA data of HBV-related HCC, which come from the same clinical samples, were used to analyze differentially expressed miRNAs (DE-miRNAs) and differentially expressed mRNAs (DE-mRNAs) between primary tumor tissues and normal liver tissues. Overlapping genes of DE-mRNAs and potential targets of DE-miRNAs were considered as candidate genes. Then functional enrichment and hub gene analysis were carried out. Crucial miRNA-mRNA axes were constructed according to hub genes and corresponding miRNAs. Furthermore, prognosis value and expression at protein and mRNA levels of hub genes were evaluated. The miRNA expression was detected based on The Cancer Genome Atlas (TCGA) data. The prognosis value of DE-miRNA was verified using Kaplan-Meier plotter database. Real-time PCR was utilized to confirm the expression level of crucial miRNAs and mRNAs. Finally, coexpression relationships of genes in crucial miRNA-mRNA axes were detected.

## 2. Methods

### 2.1. Data collection

Data series related to the miRNA and mRNA expression in HBV-related HCC were obtained from Gene Expression Omnibus (GEO) database (https://www.ncbi.nlm.nih.gov/geo/). The miRNA and mRNA data from the same patients were selected and data series focusing on varied HCC cell lines or animal experiments were excluded. Besides, we only recruited the data series containing more than 10 HCC samples. Finally, only two data series including GSE76903 and GSE77509 were selected for further analysis. The platform of GPL16791 (Illumina HiSeq 2500 (Homo sapiens) and GSE76903 were used to detect miRNA expression data, while GSE77509 was used to examine the mRNA expression profile. These two data series comprised the identical clinical samples of 20 primary tumor tissues and adjacent normal liver tissues from patients with HBV-related HCC 13.

### 2.2. Analysis of DE-miRNAs

The LIMMA package of R software (Version 3.5.3) was used to identify the DE-miRNAs of GSE76903. |LogFC (fold change)| > 2 and adj *p* value < 0.05 were set as criteria for screening DE-miRNAs. Volcano plot was produced using the tool of Sanger Box (http://sangerbox.com/).

### 2.3. Processing of the mRNA data and analysis of DEGs

To verify target genes of DE-miRNAs, the mRNA data from the same clinical samples was analyzed. Raw data of GSE77509 was acquired from GEO database. Quantiles was used for data standardization and R package “LIMMA” was used for data normalization. After data processing, “LIMMA” package was used to identify the DEGs. Gene ID conversation was finished in biological dataBase network (bioDBnet, https://biodbnet-abcc.ncifcrf.gov/db/db2db.php). |LogFC| > 2 and adj* p* value < 0.05 were set as criteria for screening DE-mRNAs.

### 2.4. Function enrichment for candidate target genes

Enrichr Database (http://amp.pharm.mssm.edu/Enrichr/) is a powerful tool that provides collective functions for a list of genes or proteins^14^, ^15^. Gene Ontology (GO) and Kyoto Encyclopedia of Genes and Genomes (KEGG) pathway enrichment were conducted using the Enrichr Database. Three items including biological process (BP), cellular component (CC), and molecular function (MF) were analyzed in GO annotation. *P* value< 0.05 was defined as statistically significant differences for the enrichment.

### 2.5. Construction of protein-protein interaction (PPI) network

STRING database, a web-based construction tool for functional protein association networks, was used to establish the PPI network of candidate genes 16. For one thing, PPI nodes with combined scores > 0.7 were imported into Cytoscape, visualizing the molecular interaction network. For another thing, cytohubba plugin of cytoscape software was used to identify the hub genes of PPI network. The intersection of top 10 nodes ranked by Degree, Betweenness, Closeness and BottleNeck were considered as hub genes.

### 2.6. Prognosis values, protein and mRNA expression analysis of hub genes

We validated the prognostic efficacy of mRNAs using online tool - The Kaplan-Meier plotter database (KMplot, https://kmplot.com/).The KMplot is capable to assess the effect of mRNA on survival in 21 cancer types including breast, liver, ovarian and gastric cancer. The Human Protein Atlas (www.proteinatlas.org) is a Swedish-based program started in 2003 with the aim to map all the human proteins in cells, tissues and organs using integration of various omics technologies. Protein expressions of hub genes in normal and HCC tissues were analyzed in The Human Protein Atlas 17, 18. The mRNA expression level in HCC was further verified using Gene Expression Profiling Interactive Analysis (GEPIA). GEPIA is a web server focusing on tumor and normal gene expression profiling based on the data from TCGA and Genotype-Tissue Expression (GTEx) project 19. |LogFC| > 1 and *p* < 0.05 were set as thresholds for recognition of significant gene expression.

### 2.7. Establishment of miRNA-mRNA regulatory axis

After hub genes were identified, we screened out specific miRNAs that regulated these hub genes according to the miRNA-target mRNA network that we have constructed before. MiRNA-mRNA axes were established between DE-miRNAs and hub genes. These miRNA-mRNA axes were considered as potential regulatory axes of HBV-related HCC and were further analyzed in the following experiments.

### 2.8. Expression and prognosis values analysis of miRNAs

The Encyclopedia of RNA Interactomes (ENCORI, http://starbase.sysu.edu.cn/index.php) is a platform that integrates more than 1.1 million miRNA-ncRNA, 2.5 million miRNA-mRNA, 2.1 million RBP-RNA and 1.5 million RNA-RNA interactions from multi-dimensional sequencing data 20. ENCORI allows researchers to perform the survival and differential expression analysis of miRNAs, lncRNAs, pseudogenes and mRNAs. In this study, expression levels of miRNAs were identified in the ENCORI online software. The Kaplan-Meier plotter (KM plotter), an online database integrating gene expression information and survival data of several cancers based on GEO database 21, was used to perform prognosis analysis of miRNAs in HCC in our study and index results of *p* < 0.05 were regarded as significant statistically.

### 2.9. Real-time PCR (RT-PCR)

HepG2-HBx, an HBV associated HCC cell line, was established in our previous study 22, and the normal hepatic cell line HL-7702 were obtained from the Cell Bank of the Chinese Academy of Sciences (Shanghai, China). RT-PCR analysis was performed to analyze the expression of miRNAs and mRNAs. To be brief, total RNA was extracted from HepG2-HBx and HL-7702 using TRIzol reagent (Invitrogen, Carlsbad, CA, U.S.). The miRNA and mRNA reverse transcriptions were performed according to the manufacturer's instructions using The One Step PrimeScript® miRNA cDNA Synthesis Kit (Takara, Japan) and PrimeScript® RT Master Mix Perfect Real Time (Takara, Japan), respectively. The miRNA and mRNA expression quantifications were carried out using SYBR® Premix Ex TaqTM II (Perfect Real Time, Takara, Japan). Expression signals were detected by ABI Quant Studio 7 Flex Real-Time PCR System. The miRNA quantifications were normalized to U6 snRNA whereas mRNA quantifications were normalized to β-actin. Primers were shown in Additional File 1: Table S1.

### 2.10. Relationship verification of screened miRNAs and mRNAs

As mentioned in Part 2.8, accompanied with gene expression data of 32 types of cancers which are derived from 10,882 RNA-seq and 10,546 miRNA-seq data, ENCORI also provides platforms for Pan Cancer analysis on RNA-RNA and RBP-RNA interactions. In our study, co-expression relationship between screened miRNA and mRNA were analyzed by Pearson correlation coefficient (*r*) in ENCORI. A negative correlation coefficient (*r* < 0) and the *p* value lower than 0.05 describe a statistically negative correlation between miRNA and mRNA.

### 2.11. Statistical analysis

All results were presented as mean ± standard deviation (SD). SPSS 17.0 software (Abbott Laboratories, Chicago, IL) was utilized for statistical analyses. The one-way analysis of variance (ANOVA) was used for statistical significance comparison among different groups. *P* < 0.05 was defined as statistical significance.

## 3. Results

### 3.1. Candidate DE-miRNAs analysis

**Figure 1:**
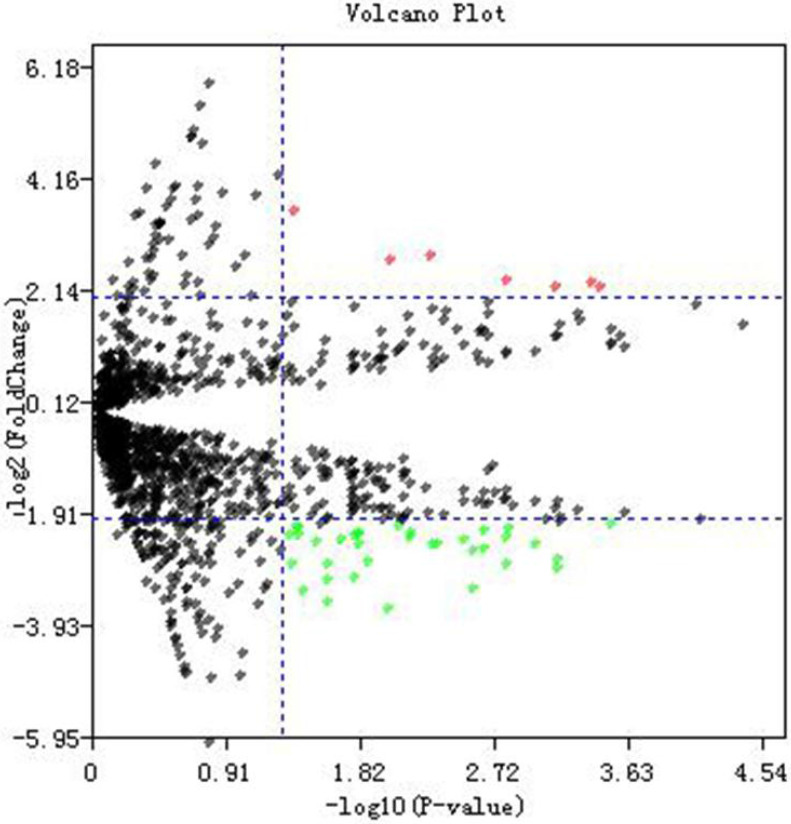
** Differentially expressed miRNAs identification.** Volcano plot of miRNA data. Red dots and green dots represent the upregulated and downregulated miRNAs in HCC samples, respectively; black dots represent miRNAs that are not differentially expressed between tumor samples and normal samples.

Dataset GSE76903 was used to identify potential DE-miRNAs between tumor samples and normal samples. A total of 2578 miRNAs were detected in GSE76903. Volcano plot of miRNA data was shown in **Fig. 1.** In accordance with the criteria we set before, and a total of 45 DE-miRNAs which includes 7 up-regulated and 38 down-regulated miRNAs were selected (Additional File 1: Table S2).

### 3.2. Identification of potential down-stream targets of DE-miRNAs

**Figure 2:**
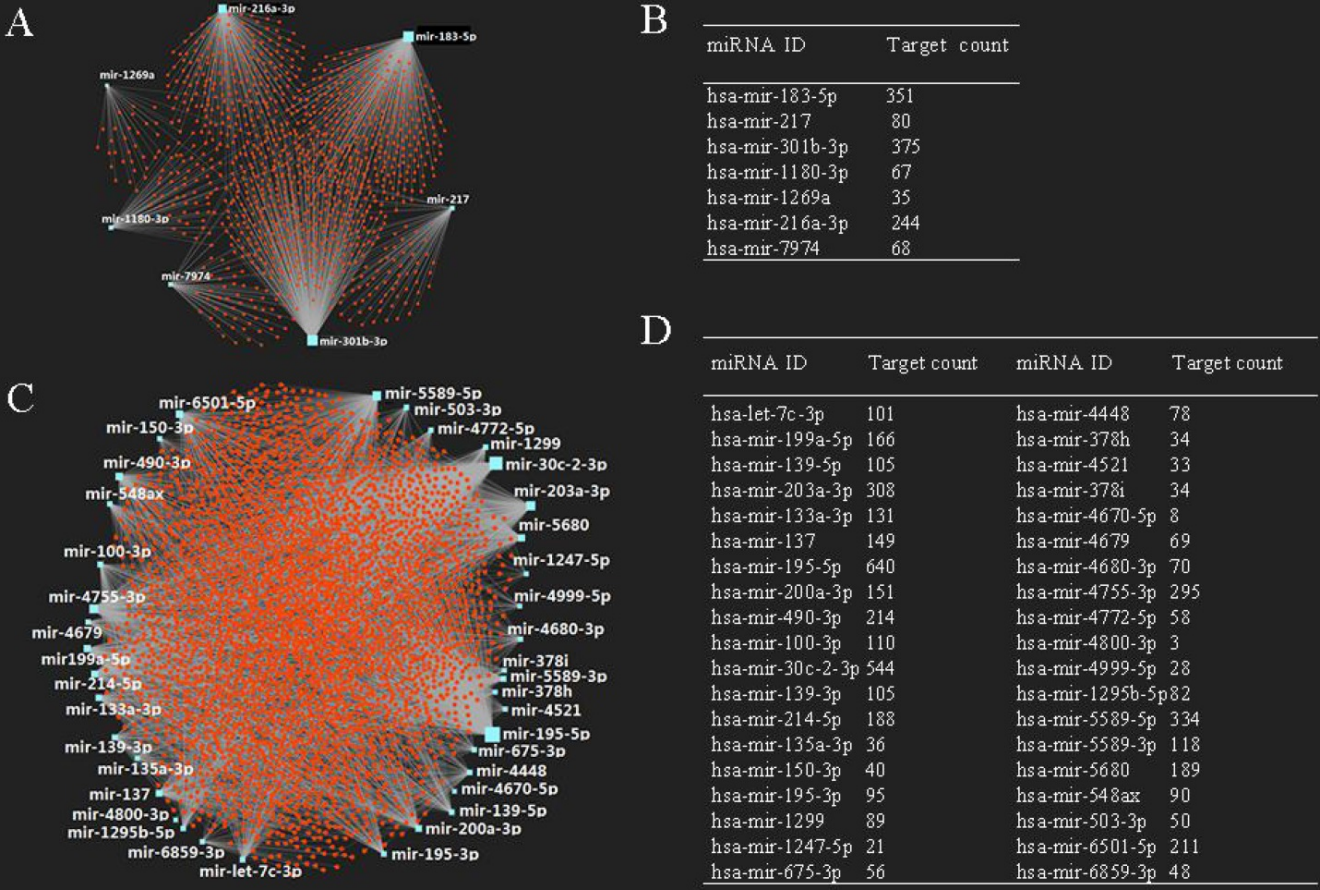
** Potential target genes of DE-miRNAs.** (A) Upregulated DE-miRNAs-target genes network; (B) downregulated DE-miRNAs-target genes network; (C) target gene count for each upregulated DE-miRNA; (D) target gene count for each downregulated DE-miRNA.

It is known that miRNA plays an important role in the regulation of mRNA. In this study, we also predicted target mRNAs of candidate DE-miRNAs using miRNet database. The results indicated that a total of 4,719 genes were obtained, including 1,103 genes for up-regulated miRNAs and 3,616 genes for down-regulated miRNAs, respectively. The construction of miRNA-target mRNA network was illustrated in Fig**. 2A, 2C**, while the target numbers of each DE-miRNAs were listed in **Fig. 2B, 2D.**

### 3.3. Identification of candidate target genes

**Figure 3:**
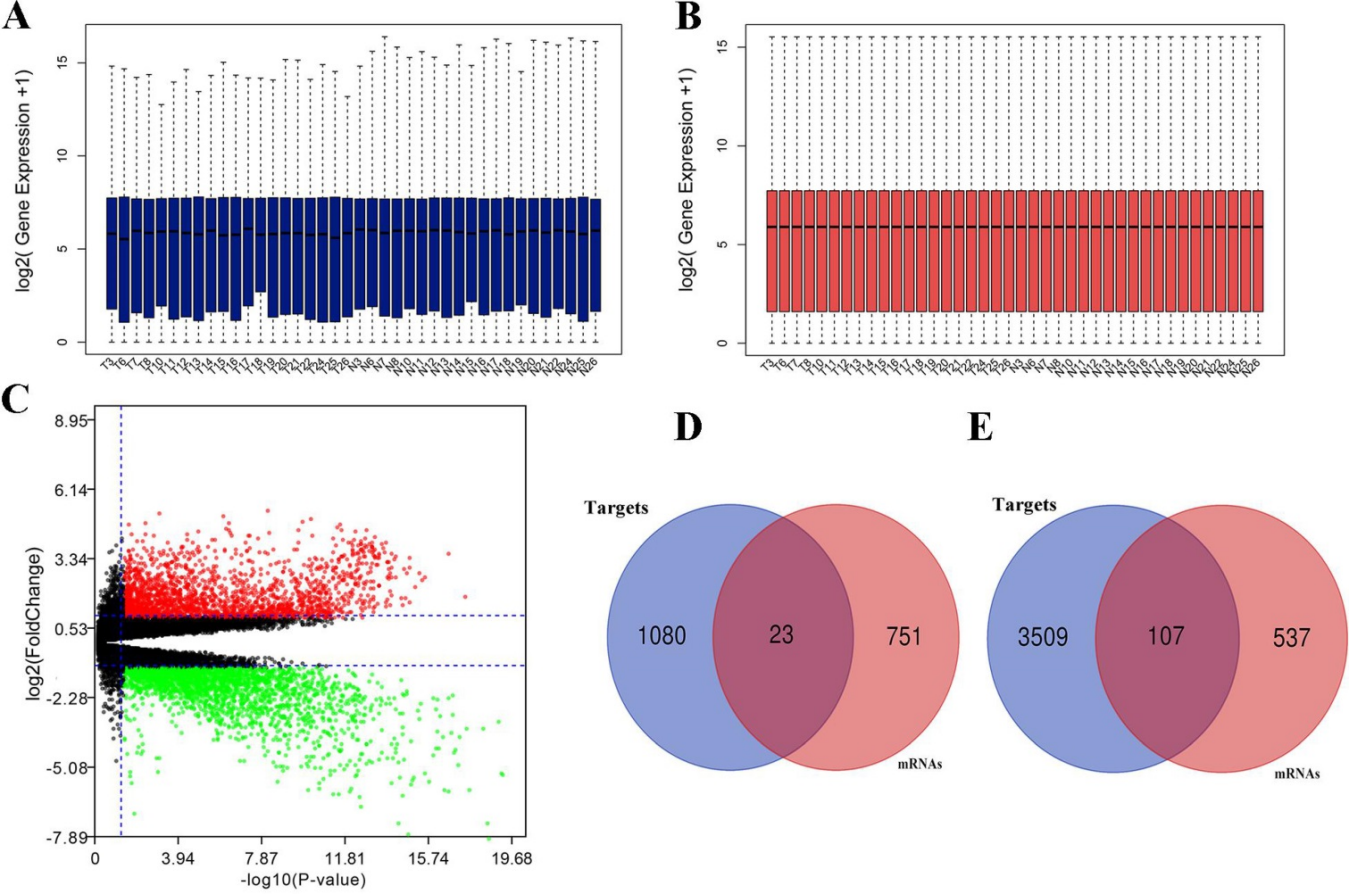
** Differentially expressed mRNAs identification.** (A) mRNA data before normalization; (B) mRNA data after normalization; (C) Volcano plot of mRNA data.; (D) the intersection of target genes of upregulated DE-miRNAs and downregulated mRNAs; (E) the intersection of target genes of downregulated DE-miRNAs and upregulated mRNAs. Red dots and green dots represent the upregulated and downregulated mRNAs in HCC samples, respectively; black dots represent mRNAs that are not differentially expressed between tumor samples and normal samples.

Expression between miRNA and target mRNA has a reverse relationship. With the obtained mRNA data from the identical clinical samples in GEO database, data standardization and normalization were performed first (**Fig. 3A, 3B**), and volcano plot of mRNA data was shown in **Figure 3C.** A total of 1,418 DEGs were identified including 644 up-regulated genes and 774 down-regulated genes based on the threshold. Subsequently, we obtained the intersections of DE-mRNAs and target genes of DE-miRNAs. As shown in **Fig. 3D, 3E**, 23 target genes of up-regulated miRNAs and 107 target genes of down-regulated miRNAs were identified as candidate genes for further analysis.

### 3.4. GO annotation enrichment for candidate target genes

**Figure 4:**
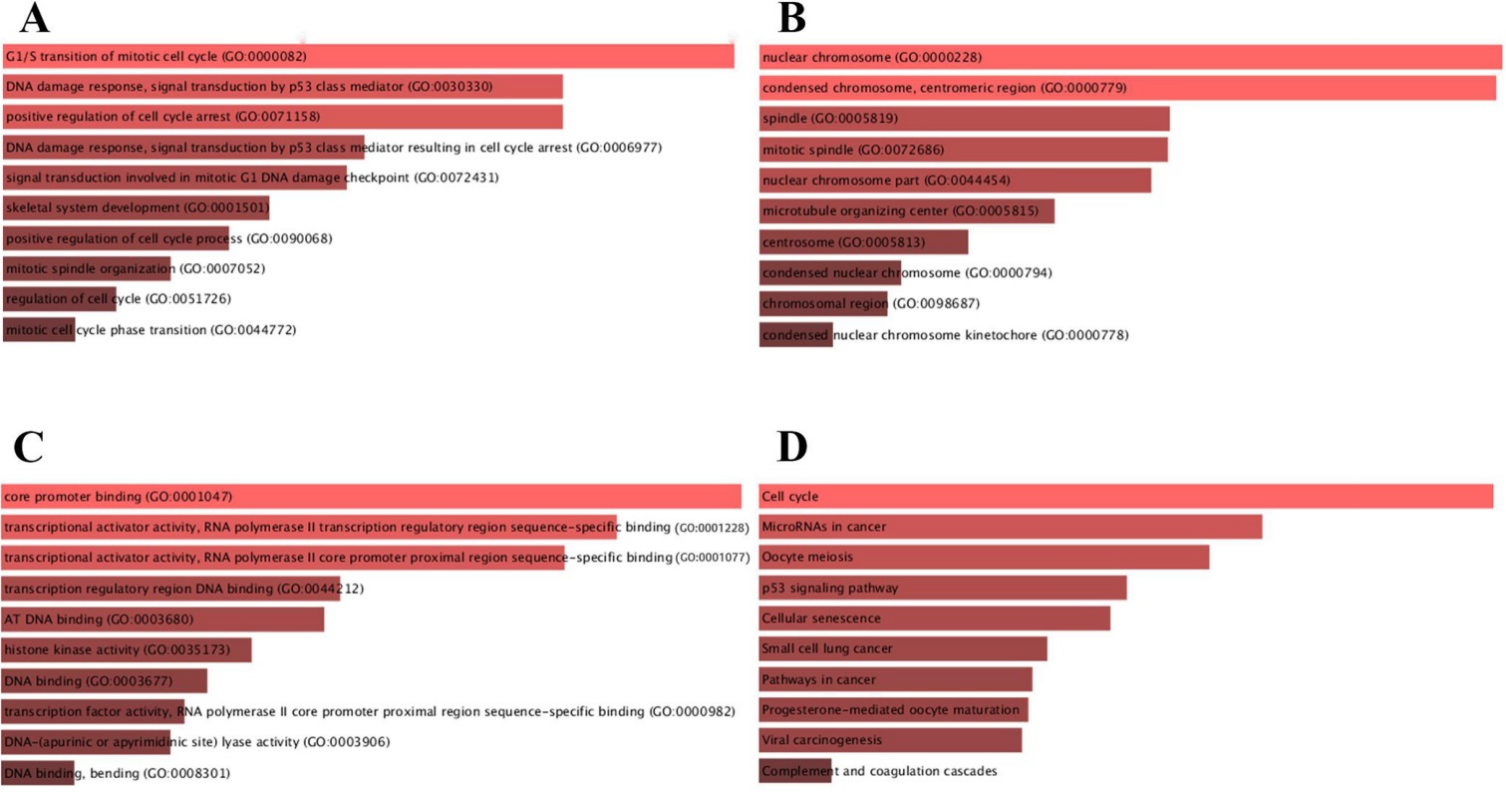
** Functional enrichment for candidate target genes.** (A) The top 10 enriched BP items of candidate genes; (B) the top 10 enriched CC items of candidate genes; (C) the top 10 enriched MF items of candidate genes; (D) The top 10 enriched KEGG pathways for candidate genes.

For deeper insights in biological functions, candidate target genes were subjected to the Enrichr database. BP, CC and MF were analyzed in GO annotation (**Fig. 4**). BP analysis suggested that candidate target genes were mainly enriched in G1/S transition of mitotic cell cycle, DNA damage response and signal transduction by p53 class mediator (**Fig. 4A**). CC analysis showed that mRNAs were significantly enriched in nuclear chromosome, condensed chromosome and centromeric region (**Fig. 4B**). MF results indicated that candidate target genes were mainly enriched in core promoter binding and transcriptional activator activity and RNA polymerase II transcription regulatory region sequence-specific binding (**Fig. 4C**). We further identified the KEGG pathway and found that enriched pathways for most of the genes were cell cycle and microRNAs in cancers as shown in **Fig. 4D.**

### 3.5. Hub genes screening from PPI network

**Figure 5:**
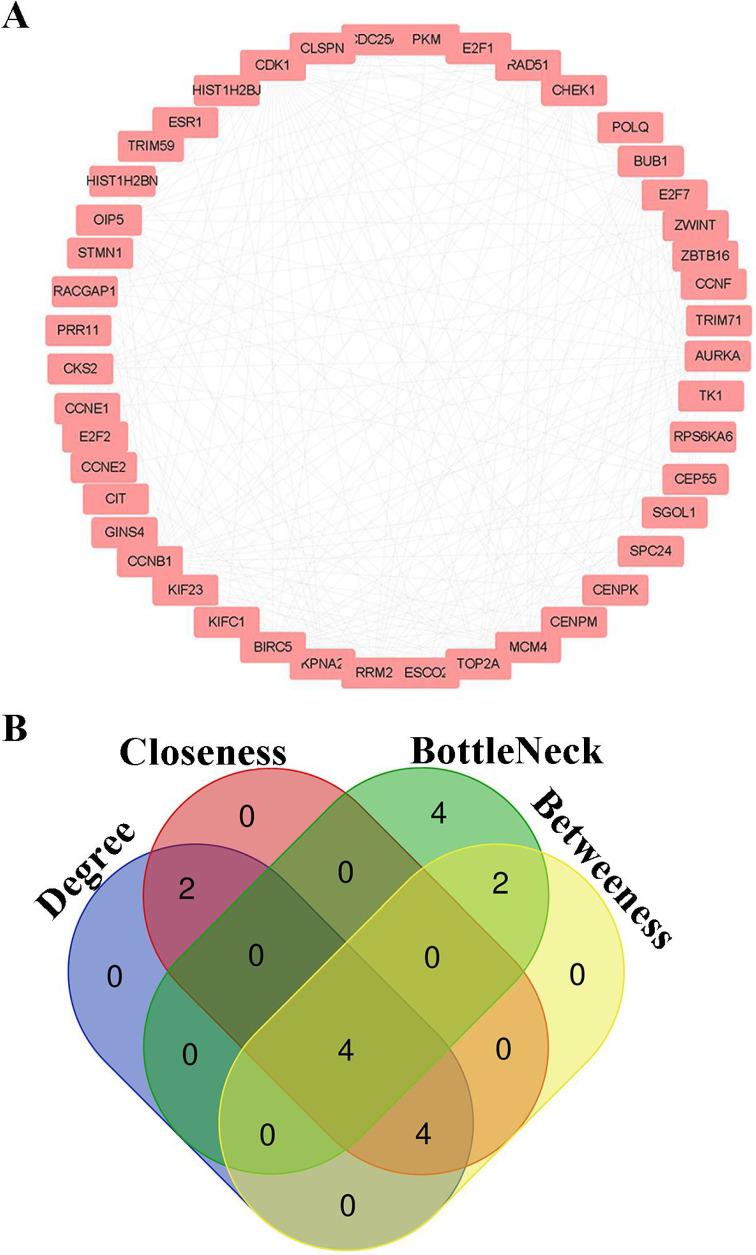
** Hub genes screening for HCC.** (A) PPI networks for candidate genes; (B) The intersection of top 10 nodes ranked by Degree, Betweenness, Closeness, BottleNeck.

PPI information of candidate genes was analyzed by String Database. Node pairs with combined score > 0.7 were imported into cytoscape to visualize PPI network (**Fig. 5A**) and screen hub genes. *CDK1, CCNB1, CKS2* and *CCNE1* were selected as hub genes according to the interaction of top 10 nodes ranked by Degree, Betweenness, Closeness, and BottleNeck (**Fig. 5B**, Additional File 1: Table S3).

### 3.6. Prognosis values, protein and mRNA expression analysis of hub genes

**Figure 6:**
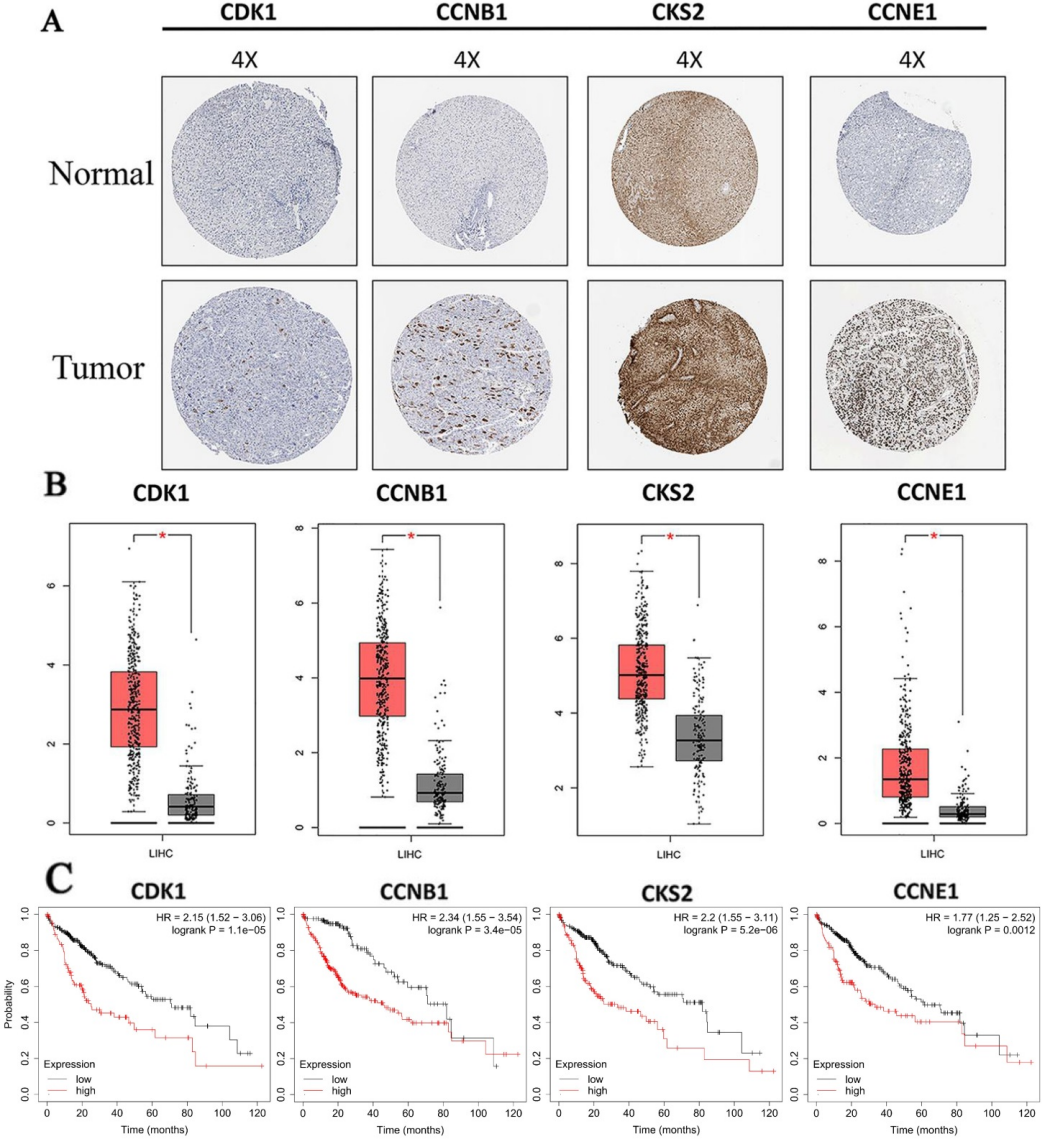
** Expression level and prognosis value of hub genes.** (A) Protein expressions of hub genes were up regulated in HCC tissues. Images were taken from the Human Protein Atlas (http://www.proteinatlas.org) online database; (B) The mRNA expressions of hub genes were up regulated. Data were taken from the GEPIA database. (C) Kaplan-Meier analysis of hub genes for patients with HCC. Red boxes and grey boxes represent the mRNA levels of tumor and nomal tissue, respectively. *P < 0.05.

Protein expressions of hub genes were analyzed to confirm the clinical relevance with HCC. Results showed that all proteins we screened were strongly expressed in HCC tissue, and weakly or negatively expressed in normal tissue (**Fig. 6A**). Furthermore, we also found that mRNA expressions of all hub genes were significantly increased in HCC compared with normal liver tissues based on TCGA data (**Fig. 6B**). In addition, the result of prognosis prediction indicated that prognosis value of hub genes were significantly different in overall survival of HCC (**Fig. 6C**). HRs and *P*-values were shown for each survival analysis. Besides, upregulation of mRNAs (all hub mRNAs were upregulated in our analysis before) were related to the poorer prognosis as we expected.

### 3.7. miRNA-mRNA regulatory axis of HBV-related HCC

After hub genes were obtained from PPI network, we constructed the preliminary miRNA-hub target genes axes based on the miRNA-mRNA pairs we have predicted before. Critical miRNA-hub target genes axes including hsa-mir-195-5p/*CDK1*, hsa-mir-5589-3p/*CCNB1*, hsa-let-7c-3p/*CKS2*, hsa-mir-195-5p*/CCNE1* and hsa-mir-30c-2-3p /* CCNE1* were acquired afterwards.

### 3.8. Expression and prognosis values analysis of miRNAs and relationship verification of screened miRNA and mRNA

**Figure 7:**
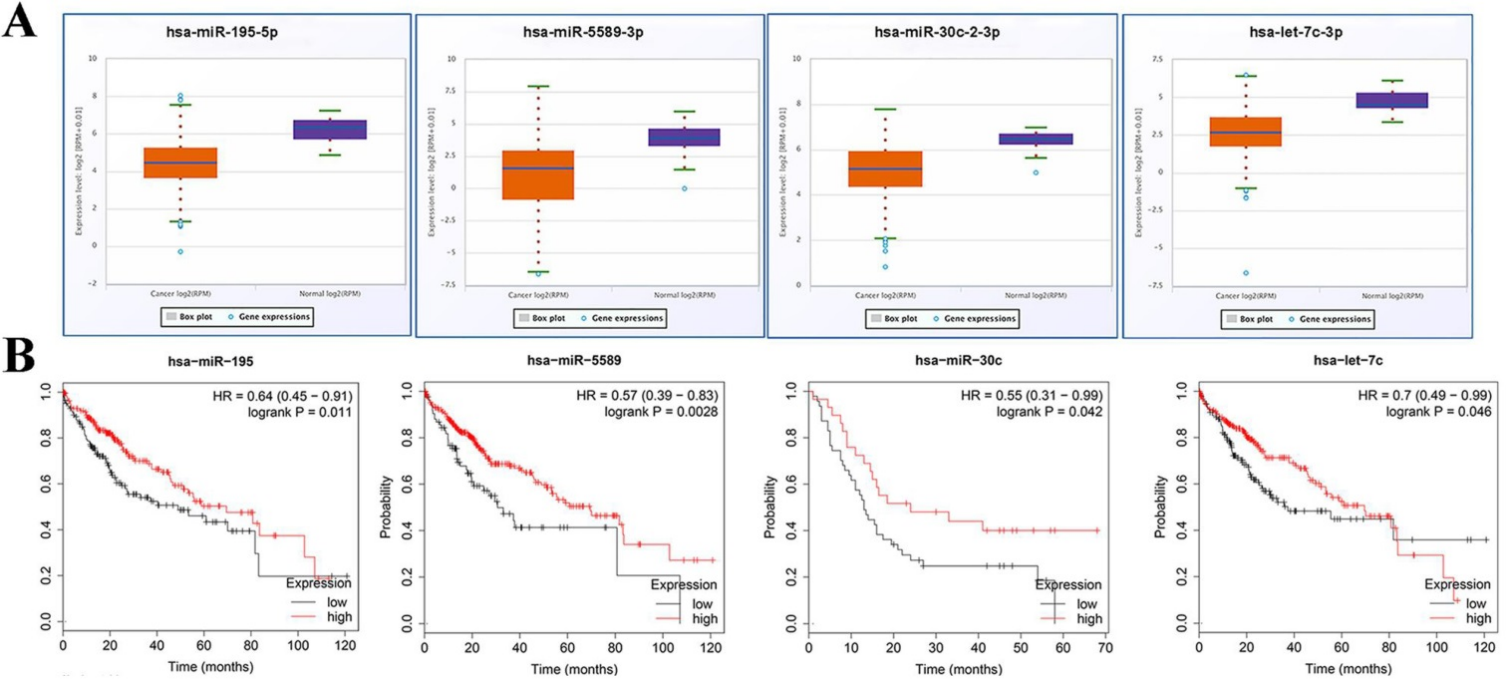
** Expression and prognosis values analysis of miRNAs.** (A) Expressions of crucial miRNAs were down regulated in HCC. (B) Downregulation of all crucial DE-miRNAs showed a significantly unfavorable overall survival rate in patients with HCC. Orange boxes and pruple boxes represent the miRNA levels of tumor and nomal tissue, respectively. *P < 0.05.

Expression of miRNA in miRNA-hub target genes axes was verified using starBase v3.0. Expectedly, all of miRNAs in preliminary axes were significantly down-regulated in TCGA data (**Fig. 7A**). Subsequently, these candidate miRNAs were typed into KM plotter database to assess their prognostic roles in HCC. As shown in **Fig. 7B**, downregulation of these DE-miRNAs illustrated a notably unfavorable overall survival rate in patients with HCC.

### 3.9. Real-time PCR

**Figure 8:**
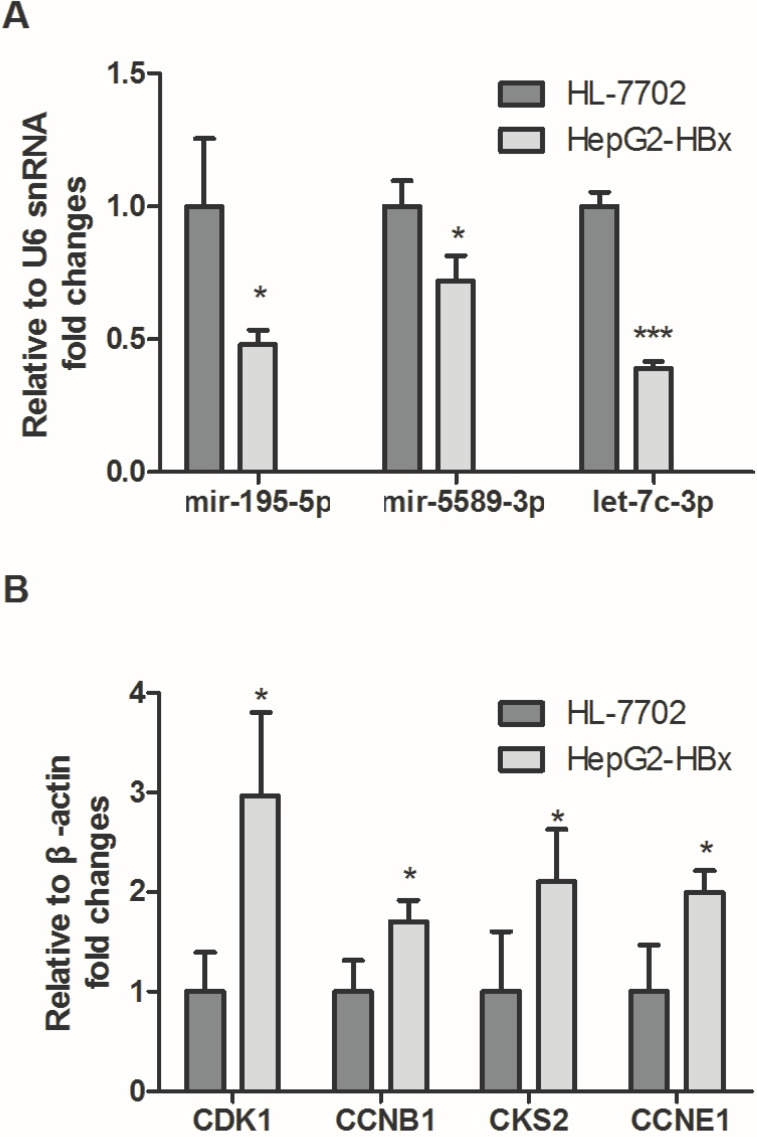
** The expression levels of crucial genes in HBV-related HCC.** (A) Expressions of crucial miRNAs were significantly downregulated in HepG2-HBx compared to normal liver cell HL-7702. (B) Expression levels of crucial mRNAs were significantly upregulated in HepG2-HBx when compared with normal liver cell.

To validate the expression of screened miRNAs and mRNAs, qPCR assay was performed in HepG2-HBx and HL-7702. In accordance with our previous analytic data, as shown in **Fig. 8A**, hsa-mir-195-5p, hsa-mir-5589-3p, hsa-let-7c-3p and hsa-mir-30c-2-3p were significantly decreased in HepG2-HBx compared with normal liver cell HL-7702. In contrast, the expression levels of four crucial targets were detected and the results showed that *CDK1, CCNB1, CKS2* and *CCNE1* were significantly increased in HepG2-HBx compared with normal liver cell (**Fig. 8B**).

### 3.10. Relationship verification of screened miRNAs and mRNAs

**Figure 9:**
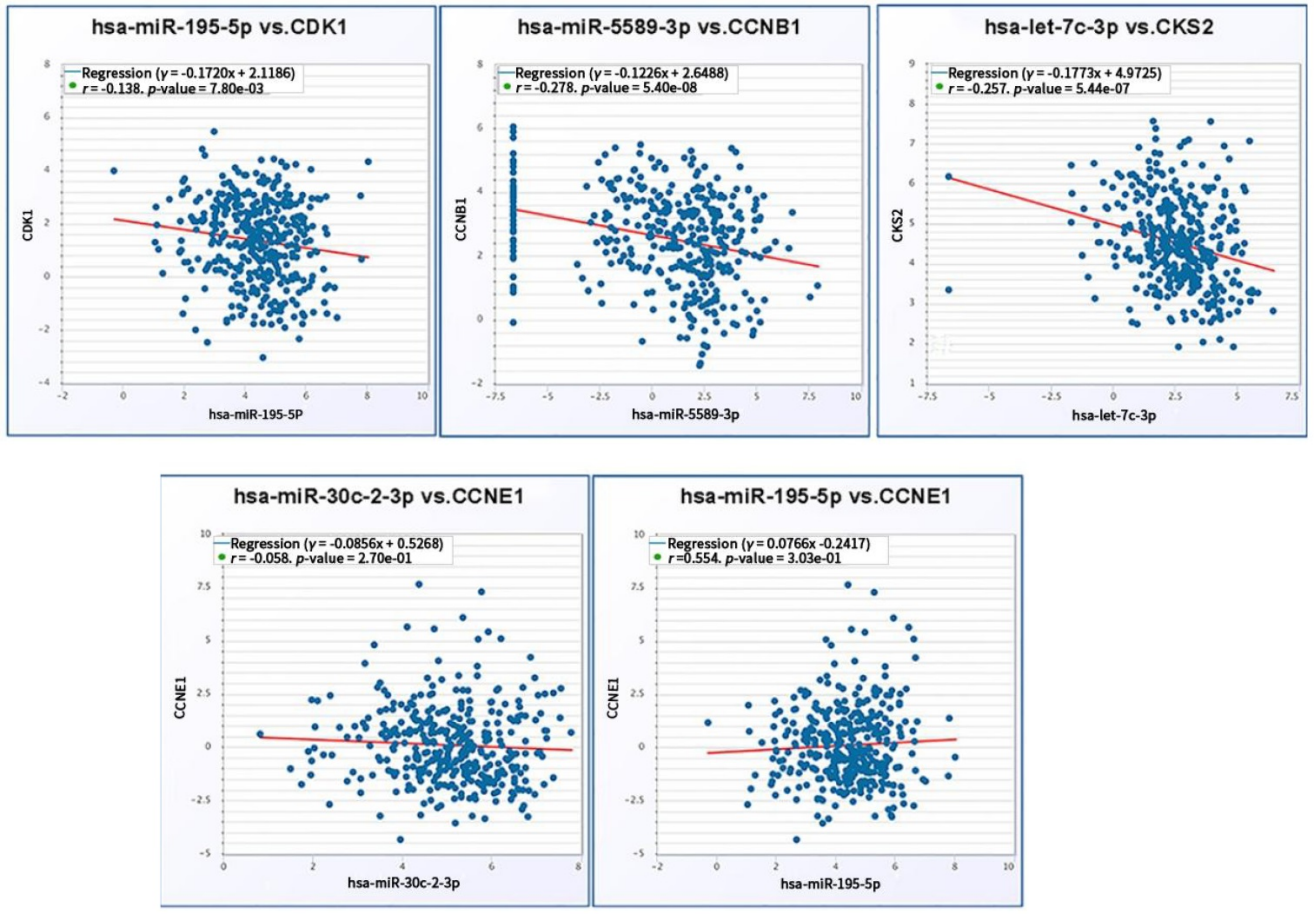
Coexpression relationship verification of screened miRNAs and mRNAs.

We further verified co-expression relationship of screened miRNAs and corresponding target mRNAs. Significant negative expression relations were validated in the axes including hsa-mir-195-5p/*CDK1* (*r* = -0.138, *p* = 7.80e-03), hsa-mir-5589-3p/*CCNB1* (*r* = -0.278, *p* = 5.40e-08) and hsa-let-7c-3p/*CKS2* (*r* = -0.257, *p* = 5.44e-07). Regarding to hsa-mir-195-5p/*CCNE1* (*r* = -0.058, *p* = 2.7e-01) and hsa-mir-30c-2-3p/*CCNE1*(*r* = 0.554, *p* = 3.03e-01), there was no significant reverse relationship between miRNA and mRNA expression (**Fig. 9**).

## 4. Discussion

In this study, differential miRNAs and mRNAs expression analysis of HBV-related HCC from the same clinical samples in GEO database were performed. 107 up-regulated and 23 down-regulated candidate genes were identified from the integration data of DE-mRNAs and target genes of DE-miRNAs. KEGG pathway enrichment analysis revealed that candidate genes were mainly enriched in the cell cycle and microRNAs in cancers pathways. Hub genes including *CDK1, CCNB1, CKS2* and* CCNE1* were identified through PPI network analysis. Hub genes were all increased at both mRNA and protein expression levels and upregulation of Hub genes indicated the poor prognosis. Crucial miRNA-mRNA axes including hsa-mir-195-5p/*CDK1*, hsa-mir-5589-3p/*CCNB1* and hsa-let-7c-3p/*CKS2* were filtered. Online database showed that crucial miRNAs were downregulated and significantly related to the prognosis value. Also, real-time PCR array confirmed that screened miRNAs were downregulated while screened mRNAs were upregulated in HBV-related HCC. Reverse relations between miRNA and mRNA in the crucial axes were further validated.

Similar research has been carried out previously. Lou *et al.* analyze the miRNA-mRNA regulatory pathways of HBV-related HCC and screen six candidate target genes via qRT-PCR validation 12. However, dataset GSE69580 selected in their study contained only 5 HBV-related HCC tumor tissues and 5 non-tumor liver tissues. Only targets of miRNAs were used for screening candidate genes regulated by DE-miRNAs. Moreover, protein expression and survival data of critical genes were not mentioned. For further research in potential miRNA-mRNA regulatory axes, we selected the miRNA and mRNA datasets from the same clinical samples which recruited 20 primary tumor tissues and 20 normal liver tissues. Overlapping genes of DE-mRNAs and target genes of DE-miRNAs were selected as candidate genes. In addition, we further analyzed the protein expression and survival data of identified core genes.

For the moment, few comprehensive miRNA-mRNA regulatory networks were identified in HBV-related HCC based on the same clinical sample. Therefore, we assessed the differential expression data of miRNA and mRNA from GEO database. We have screened 7 up-regulated and 38 down-regulated DE-miRNAs in HBV-related HCC samples compared with normal tissues. It is worth noticing that previous researches have shown that the majority of expressions of the DE-miRNAs are consistent with our analysis. For instance, hsa-miR-1269a is up-regulated and might functions as an onco-miRNA 23. MiR-217 is a factor promoting cancer stem cell properties via activation of the *DKK1*/Wnt signaling pathway in HCC 24. Hsa-miR-139-5p, which probably act as tumor suppressors, is decreased and associated with 3-year overall survival of HCC patients 23. Study shows that hsa-miR-139-5p also exhibits cell growth inhibition through targeting *SPOCK1 in vitro* 25. MiR-137 is decreased in HCC and inhibited migration and invasion via targeting *EZH2-STAT3* axis in human HCC 26, 27.

In this study, we further assessed the DE-mRNAs and target genes of DE-miRNAs. From the overlapping genes between DE-mRNAs and target genes of DE-miRNAs, 107 up-regulated and 23 down-regulated mRNAs were screened as candidate genes. Pathway enrichment detection showed that the cell cycle and microRNAs in cancers were the crucial pathway for candidate genes. Malignant tumors are characterized by uncontrolled cell proliferation. Disorder genes controlling cell cycles such as *p16INK4a* and *cyclin Dl*, abnormal proteins regulating phosphorylation of the retinoblastoma protein (RB) and exiting from the G1 phase are frequently detected in dysfunctional malignant tumor cells 28. Signaling pathways of most proto-oncogenes and tumor suppressor genes are focused on the GC phase, GI cyclins and *CDK1* regulation, therefore interfering progression and checkpoint of the cell cycle 28. With integration into growth essential genes (such as *TERT*), pro-oncogenes and tumor suppressor genes, HBV promote hepatocarcinogenesis through the carcinogenic character of truncated HBx gene by accelerating G1/S and G2/M transition 29-31.

The hub genes including* CDK1, CCNB1, CKS2* and *CCNE1* were screened from PPI network analysis. According to TCGA data, expressions of the above mentioned hub genes were higher in HCC. Prognosis value data showed that overexpression of *CDK1, CCNB1, CKS2* and* CCNE1* heralded a worse outcome. *CDK1,* a serine/threonine kinase, is related to the eukaryotic cell cycle and mitogenesis. Specifically, it accelerates the G2-M transition and controls G1 progression and G1-S transition by binding to varies of interphase cyclins 32-35.* CCNB1* and *CKS2* are essential for the cell cycle transition. *CKS2* binds to the catalytic subunit of the cyclin dependent kinases 36. *CCNB1* forms a maturation promoting factor (MPF) (a serine/threonine kinase holoenzyme complex) through interaction to *CDK1* to promote mitosis resumption 37. *CCNE1* plays a positive role in cell cycle progression through regulating the transition of G1/S phase 38. In line with pathway enrichment analysis, all hub genes were closely related to the cell cycle process. Therefore, *CDK1, CCNB1, CKS2* and* CCNE1* are supposed to be increased in HBV-related HCC. Previous research demonstrated that HBV expression persistently activates the* CCNB1-CDK1* kinase in HCC cells 39. Chin *et al.* deliver the replicated HBV genome into a hepatocyte cell line HuH-7 to confirm that *CCNB1* is over expressed 40. Sung *et al.* found that *CCNE1* is increased in HBV-related tumors compared with adjacent normal tissue through integrating HBV sequence 41. In line with these studies, we further validated that the expression of *CDK1, CCNB* and* CCNE1* increased in HBV-related HCC via qPCR array. Overexpression of *CDK1, CCNB1* and* CCNE1* also indicated poor prognosis. Although no previous studies have given any supporting evidence directly indicating the expression and function of CKS2 in HBV-related HCC, some researchers have investigated its relationship with HCC and viral infection-induced woodchuck HCC.* CKS2* is overexpressed in HCC at both mRNA and proteins levels and upregulates in woodchuck HCC at mRNA level 36 and its overexpression is related to poor differentiation and the aggressive behaviour of HCC 36. For the very first time, our study raises the idea that *CKS2* is expressed at a higher level and might results in poor prognosis in HBV-related HCC.

The inhibition mechanism of miRNA is a complex interaction between miRNA and mRNA. The down-regulated miRNAs might contribute to the upregulation of genes related to cell cycle through binding to the 3-untranslated region (UTR). Our study has analyzed the DE-miRNAs of HBV-related HCC and investigated the intersection genes between their target genes and DE-mRNAs. Expression validation showed that all miRNAs were decreased in HBV-related HCC. However, co-expression analysis indicated that only three axes including hsa-mir-195-5p/*CDK1*, hsa-mir-5589-3p/*CCNB1* and hsa-let-7c-3p/*CKS2* had the inverse relationship between miRNA and target genes. Won *et al*. find that the serum level of exosomal miR-195 is lower in patients with HCC and suggest that miR-195 could be a novel serological biomarker for HCC 42. Xu *et al*. demonstrated that miR-195 could reduce SETD3 at post transcription level and therefore be associated with promoting proliferation of HCC 43. Furuta *et al*. screen crucial tumor-suppressive miRNAs in HCC, and find that miR-195 turns out to be frequently silenced in HCC and performs significant growth-inhibitory activity via induction of G1 arrest 44. Pathway analysis and target exploration of miR195 demonstrate a significant enrichment of genes related to cell cycle regulation. Through transfection of mimicking dsRNA of miR195 into HCC cell lines, they find *CCNE1* and *CDC25A* may be novel direct target gene for miR195 44. Hsa-let-7c-3p is reported to be one of the targets of multiple circular RNAs which promotes skin cancers and progression of noncancerous diseases 43. For the time being, though, there are no reports revealing the expression and function of miR-5589. Hence, we preliminarily verified lower expression of miR-5589 and hsa-let-7c-3p in HBV-related HCC. Therefore, this study may firstly put forward the correlation between HBV-related HCC and miR-5589/hsa-let-7c-3p, providing some novel visions for future research on relevant mechanisms.

Although an integrated bioinformatics analysis has been conducted and potential miRNA-mRNA regulatory axes have been established in our study. Inevitably, some limitations still exist. Resulting from the deficient clinical data from GEO dataset, we could only make a hypothesis of the prognosis value of mRNAs identified based on TCGA data. The expressions of certain mRNAs and miRNAs have not been further confirmed through *in vivo* experiments and their roles in genesis, development and metastasis of cancer as well as HBV infection are worth studying in future.

## 5. Conclusions

In summary, we analyzed the potential regulatory miRNA-mRNA axes of HBV-related HCC by means of various bioinformatics tools. Hsa-mir-195-5p/*CDK1*, hsa-mir-5589-3p/*CCNB1* and hsa-let-7c-3p/*CKS2* are likely to be the crucial axes involved in the genesis and development of HBV-related HCC with diagnostic and prognostic value. Furthermore, they are potential to be the novel therapeutic targets for HBV-related HCC. Further studies and experiments are needed in order to validate the potential roles of predictive miRNA-mRNA axes in HBV-related HCC.

## Supplementary Material

Supplementary tables.

## Abbreviations

HBV: Hepatitis B virus
HCC: Hepatocellular carcinoma
DE-miRNAs: Differentially expressed miRNAs
DE-mRNAs: Differentially expressed mRNAs
TFs: Transcription factors
PPI: Protein-Protein Interaction
TCGA: The Cancer Genome Atlas
HCV: Hepatitis C virus
GEO: Gene Expression Omnibus database
LogFC: Log (fold change)
GO: Gene Ontology
KEGG: Kyoto Encyclopedia of Genes and Genomes
BP: Biological process
CC: Cellular component
MF: Molecular function
GEPIA: Gene Expression Profiling Interactive Analysis
GTEx: Genotype-Tissue Expression
Kaplan-Meier plotter: KM plotter
MPF: maturation promoting factor
