# Supplementary material for: Bioinformatics Analyses of Potential miRNA-mRNA Regulatory Axis in HBV-related Hepatocellular Carcinoma
Source: Int J Med Sci. 2021 Jan 1;18(2):335–46. doi: 10.7150/ijms.50126 (PMC7757140; doi:10.7150/ijms.50126)
Supplement: Supplementary file 1 — Supplementary tables. [file ijmsv18p0335s1.pdf]

Supplement Table 1. Primers used for quantitative real-time PCR in this study.

Name	Direction	Primer (5’-3’)
For microRNA qPCR		
Universal qPCR primer	Reverse	The One Step PrimeScript® miRNA cDNA Synthesis Kit (Takara, Japan)
hsa-mir-195-5p	Forward	CGCCTAGCAGCACAGAAATATTG GC
hsa-mir-5589-3p	Forward	TGCACATGGCAACCTAGCTCC
hsa-let-7c-3p	Forward	CGCGTGTACAACCTTCTAGCTTTC C
hsa-mir-30c-2-3p	Forward	GCTGGGAGAAGGCTGTTTACTCT
U6 snRNA	Forward	CTCGCTTCGGCAGCACA
For mRNA qPCR		
CCNB1	Forward	GACTTTGCTTTTGTGACTGACA
	Reverse	CCCAGACCAAAGTTTAAAGCTC
CDK1	Forward	CACAAAACTACAGGTCAAGTGG
	Reverse	GAGAAATTTCCCGAATTGCAGT
CKS2	Forward	CCCAGAGAACTTTCCAAACAAG
	Reverse	GAATCATGTAATGAACCCAGCC
CCNE1	Forward	TTGTGTCCTGGCTGAATGTATA
	Reverse	AAGGAAATTCAAGGCAGTCAAC
β-actin	Forward	ATGTGGATCAGCAAGCAGGA
	Reverse	AAGGGTGTAAAACGCAGCTCA

Supplement Table 2. Differentially expressed miRNAs.

miRNAs	logFC	adj.P.Val	miRNAs	logFC	adj.P.Val
hsa-miR-1269a	3.65	4.53E-02	hsa-miR-203a	-2.32	3.18E-02
hsa-miR-217	2.85	5.51E-03	hsa-miR-1247-5p	-2.35	5.21E-03
hsa-miR-216a-3p	2.76	5.51E-03	hsa-miR-199a-5p	-2.36	4.96E-03
hsa-miR-183-5p	2.41	1.67E-03	hsa-miR-133a-3p	-2.37	1.05E-03
hsa-miR-1180-3p	2.36	4.49E-04	hsa-miR-4448	-2.37	1.69E-02
hsa-miR-301b	2.30	3.86E-04	hsa-miR-214-5p	-2.46	2.33E-03
hsa-miR-7974	2.27	7.71E-04	hsa-miR-675-3p	-2.51	2.80E-03
hsa-miR-195-5p	-2.02	3.23E-04	hsa-miR-30c-2-3p	-2.54	2.88E-05
hsa-miR-503-3p	-2.03	4.44E-02	hsa-miR-139-3p	-2.67	7.39E-04
hsa-miR-4521	-2.04	9.02E-03	hsa-miR-4755-3p	-2.69	1.43E-02
hsa-miR-1299	-2.08	4.28E-02	hsa-miR-4679	-2.73	2.72E-02
hsa-miR-1295b-5p	-2.10	1.60E-03	hsa-miR-4800-3p	-2.73	1.67E-03
hsa-miR-4999-5p	-2.13	2.33E-03	hsa-miR-4680-3p	-2.75	4.69E-02
hsa-miR-150-3p	-2.15	1.66E-02	hsa-miR-139-5p	-2.82	2.88E-05
hsa-miR-195-3p	-2.15	7.49E-03	hsa-miR-6501-5p	-2.82	7.39E-04

hsa-miR-378i	-2.19	1.69E-02	hsa-miR-5589-5p	-2.97	1.77E-02
hsa-miR-135a-3p	-2.22	4.87E-02	hsa-miR-4670-5p	-3.01	2.76E-02
hsa-miR-5589-3p	-2.22	4.34E-02	hsa-miR-490-3p	-3.17	2.87E-03
hsa-miR-4772-5p	-2.23	1.78E-02	hsa-miR-6859-3p	-3.24	3.97E-02
hsa-miR-100-3p	-2.24	7.57E-03	hsa-miR-137	-3.42	2.73E-02
hsa-let-7c-3p	-2.25	1.65E-03	hsa-miR-548ax	-3.55	1.07E-02
hsa-miR-378h	-2.28	2.19E-02	hsa-miR-5680	-3.96	2.88E-05
hsa-miR-200a-3p	-2.31	3.29E-03			

Supplement Table 3. Hub genes screening using cytohubba.

Gene	Degree	Gene	Betweenness	Gene	BottleNeck	Gene	Closeness	Overlapped	Expression
CDK1	15	CDK1	122.3	CDK1	10	CDK1	16.5	CDK1	UP
CCNB1	13	CCNB1	54.9	CCNB1	7	CCNB1	15.33	CCNB1	UP
OIP5	8	HIST1H2BJ	34	CKS2	4	OIP5	12.67	CKS2	UP
CKS2	7	CCNF	34	HIST1H2BJ	2	CKS2	12.333	CCNE1	UP
MCM4	7	CCNE1	18.9	CCNF	2	MCM4	12.167		
CHEK1	6	CKS2	16.4	CCNE1	2	CDC25A	11.67		
CDC25A	6	CCNE2	15.4	E2F7	1	CCNE1	11.67		
KIF23	6	OIP5	8.9	CENPK	1	CCNE2	11.67		
CCNE1	6	MCM4	4.4	IFITM1	1	CHEK1	11.5		
CCNE2	6	CDC25A	2.9	EGR1	1	KIF23	11.5		
